# Supplementary material for: Combining Materials Obtained by 3D-Printing and Electrospinning from Commercial Polylactide Filament to Produce Biocompatible Composites
Source: Polymers (Basel). 2021 Nov 3;13(21):3806. doi: 10.3390/polym13213806 (PMC8588263; doi:10.3390/polym13213806)
Supplement: Supplementary file 1 [file polymers-13-03806-s001.zip › polymers-1439640-supplementary.pdf]

Supplementary Material for the article:
Combining PLA based materials obtained by 3D-printing and electrospinning to produce biocompatible scaffolds
From Pablo Romero-Araya, et al.

1. Hydroxyapatite synthesis.

HA was synthesized using the sol-gel precipitation method reported in literature [1,2]. Briefly, 100 mL of 0.6 M potassium dihydrogen phosphate (KH_2PO_4) solution was mixed dropwise to 100 mL of 1.0 M calcium nitrate ($Ca(NO_3)_2{\cdot}4H2O$) solution under constant magnetic stirring and at room temperature. To induce precipitation, aqueous ammonia solution was added dropwise until reach pH 11, and the resulting mixture was stirred during 1 h, and aged at room temperature during 24 h. After that, the white precipitate was recovered and subsequently washed by applying three cycles of centrifugation and redispersion in pure water. The precipitate was dried at 40 ºC during 24 h, heated to 200 ºC at a rate of 10 ºC/min, and calcined at 200 ºC during 24 h. This methodology produces less crystalline particles with needle-shape and an average size of 40 · 10 nm.

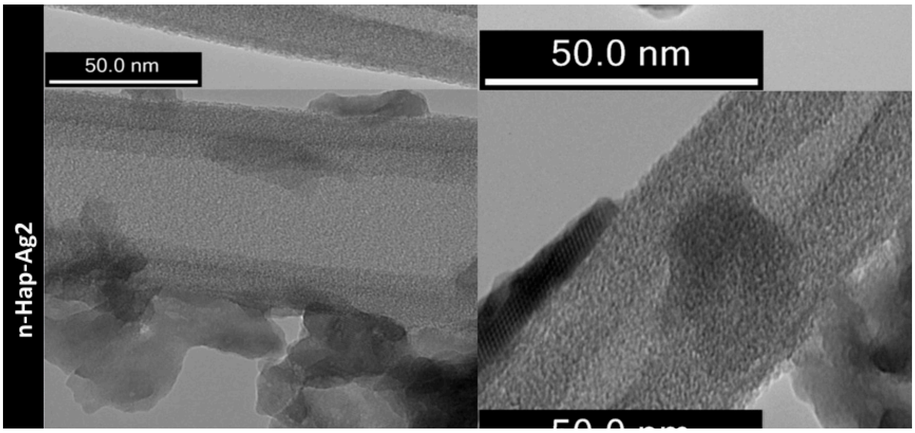

Figure S1. TEM synthesized HA nanoparticles

SEM-EDS was done to the synthesized HA. The EDS spectrum of HA was measured in a specific zone of the analyzed sample. In this scanned zone, Ca and P are found (38.8 and 16.4 wt.%, respectively). Ca/P ratio obtained is 2.37, similar value informed in literature for HA obtained from biowaste (2.32) [3].

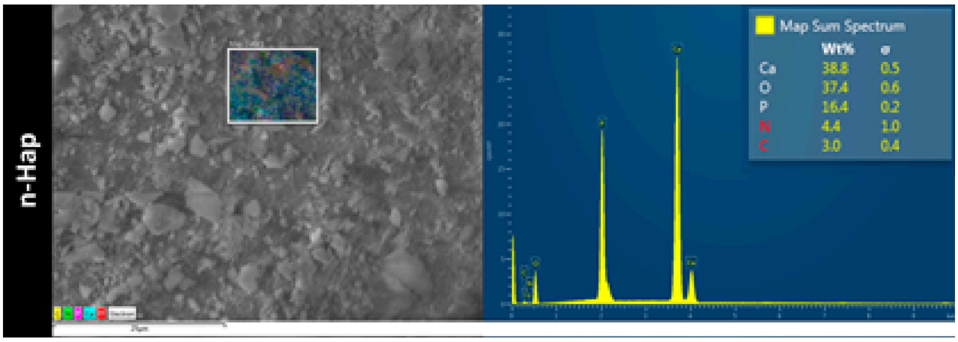

Figure S2. SEM-EDS analysis of synthesized HA.

2. SBF assay

Table S1. Reagents, amount and order of addition in the preparation of 1 L of SBF media.[4]

Order	Reagents	Amount (g/mL)
**1**	NaCl	7.996 g
**2**	$NaHCO_3$	0.350 g
**3**	KCL	0.224 g
**4**	$K_2HPO_4 \cdot 3H_2O$	0.228 g
**5**	$MgCL_2 \cdot 6H_2O$	0.305 g
**6**	1M-HCl	40 mL
**7**	$CaCl_2$	0.278 g
**8**	Na_2SO_4	0.071 g
**9**	$(CH_2OH)_3CNH_2$	6.057 g

Table S2. SEM-EDS analysis of composites studied in SBF media

Sample	Ca (wt.%)	P (wt.%)	Ca/P ratio
Square composite			
PLA/PEG/2.5HA	11	2.6	5.9 ± 3.1
PLA/PEG/5.0HA	5.4	0.7	8.6 ± 3.8
Ellipsoidal composite			
PLA10	6.3	0.7	8.9 ± 2.0
PLA/PEG/2.5HA	5.8	1.1	7.4 ± 3.9

PLA/PEG/5.0HA	5.1	1.0	5.2 ± 2.7

3. References

1. Covarrubias, C.; Arroyo, F.; Balanda, C.; Neira, M.; Von Marttens, A.; Caviedes, P.; Rodríguez, J.P.; Urra, C. The Effect of the Nanoscale Structure of Nanobioceramics on Their *In Vitro* Bioactivity and Cell Differentiation Properties. *J. Nanomater.* **2015**, *2015*, 526230, doi:10.1155/2015/526230.
2. Sanosh, K.P.; Chu, M.-C.; Balakrishnan, A.; Lee, Y.-J.; Kim, T.N.; Cho, S.-J. Synthesis of nano hydroxyapatite powder that simulate teeth particle morphology and composition. *Curr. Appl. Phys.* **2009**, *9*, 1459–1462, doi:https://doi.org/10.1016/j.cap.2009.03.024.
3. Abifarin, J.K.; Obada, D.O.; Dauda, E.T.; Dodoo-Arhin, D. Experimental data on the characterization of hydroxyapatite synthesized from biowastes. *Data Br.* **2019**, *26*, 104485, doi:https://doi.org/10.1016/j.dib.2019.104485.
4. Kokubo, T.; Takadama, H. How useful is SBF in predicting in vivo bone bioactivity? *Biomaterials* **2006**, *27*, 2907–2915, doi:10.1016/j.biomaterials.2006.01.017.
